# Vaccinia virus injected human tumors: oncolytic virus efficiency predicted by antigen profiling analysis fitted boolean models

**DOI:** 10.1080/21655979.2019.1622220

**Published:** 2019-05-29

**Authors:** Alexander Cecil, Ivaylo Gentschev, Marion Adelfinger, Thomas Dandekar, Aladar A. Szalay

**Affiliations:** a Department of Biochemistry, Theodor-Boveri-Institute, University of Würzburg, Biocenter, Würzburg, Germany; b Department of Bioinformatics, Theodor-Boveri-Institute, University of Würzburg, Biocenter, Würzburg, Germany; c Genelux Corporation, San Diego, CA, USA; d Department of Radiation Medicine and Applied Sciences, Rebecca & John Moores Comprehensive Cancer Center, University of California, San Diego, CA, USA

**Keywords:** Oncolytic virus, human xenografted mouse models, cancer therapy, boolean modeling

## Abstract

Virotherapy on the basis of oncolytic vaccinia virus (VACV) strains is a promising approach for cancer therapy. Recently, we showed that the oncolytic vaccinia virus GLV-1h68 has a therapeutic potential in treating human prostate and hepatocellular carcinomas in xenografted mice. In this study, we describe the use of dynamic boolean modeling for tumor growth prediction of vaccinia virus-injected human tumors. Antigen profiling data of vaccinia virus GLV-1h68-injected human xenografted mice were obtained, analyzed and used to calculate differences in the tumor growth signaling network by tumor type and gender. Our model combines networks for apoptosis, MAPK, p53, WNT, Hedgehog, the T-killer cell mediated cell death, Interferon and Interleukin signaling networks. The *in silico* findings conform very well with *in vivo* findings of tumor growth. Similar to a previously published analysis of vaccinia virus-injected canine tumors, we were able to confirm the suitability of our boolean modeling for prediction of human tumor growth after virus infection in the current study as well. In summary, these findings indicate that our boolean models could be a useful tool for testing of the efficacy of VACV-mediated cancer therapy already before its use in human patients.

## Graphical Abstract

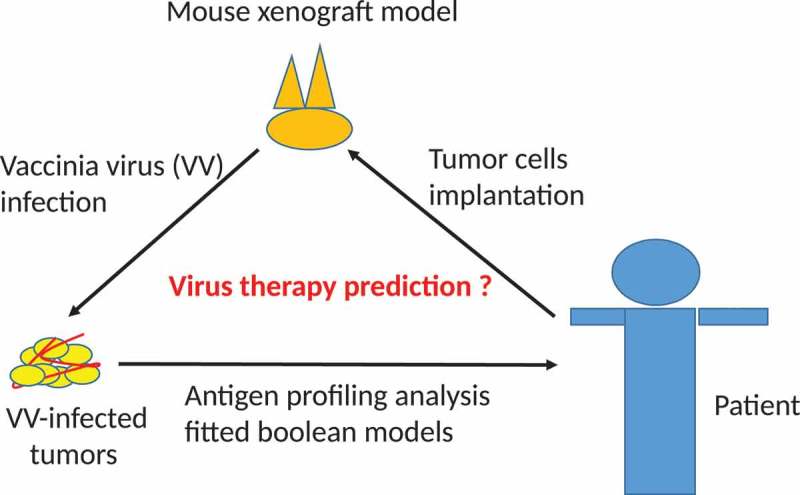

## Introduction

Cancer is the leading cause of disease-related death in humans. The major options for cancer therapy include surgery, radiation therapy and chemotherapy. However, the available treatment options for cancer patients with advanced-stage disease are limited and the prognosis for such patients is very poor. Therefore, the development of new therapies for advanced cancer has a high priority. Virotherapy using oncolytic viruses (OVs) is one promising strategy for cancer therapy. OVs preferentially infect and lyse cancer cells, without causing excessive damage to surrounding healthy tissue, and initiate tumor-specific immunity. Several oncolytic viruses including herpes simplex virus (HSV), reovirus and vaccinia virus are in or entering Phase III clinical trials (for reviews see [1–3]). In addition, in China the oncolytic adenovirus H101 has been approved in the treatment of human patients with head and neck cancer since 2005 [4]. A further milestone in the history of oncolytic virus therapy was the approval of Talimogene laherparepvec (T-VEC; Imlygic™), a genetically modified herpes simplex virus, type 1, by the US Food and Drug Administration (FDA) and the European Medicine Agency (EMA) for the treatment of advanced melanoma (Press Releases Amgen 10/27/2015 and 12/17/2015).

**Figure 2.**
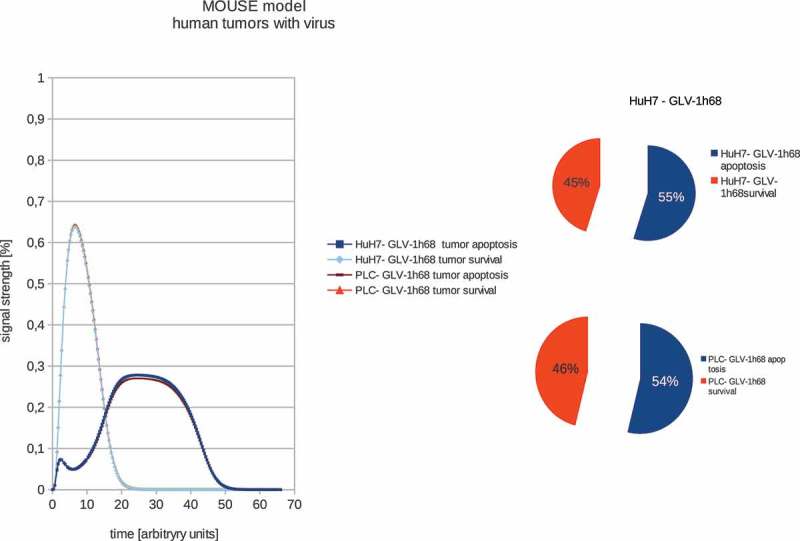
Models and ratios of tumor survival and apoptosis of the human PLC and HuH7 tumors in female nude mice after therapy with GLV-1h68 vaccinia virus strain. Again the polynomial areas of the diagrams were calculated. After virus therapy both tumor strains are moved into apoptosis.

The vaccinia virus strain GLV-1h68 (name in clinical trials GL-ONC1) was already successful used for treatment of human cancer in pre-clinical [5–7] and clinical studies Phase I and II (https://en.wiki2.org/wiki/GL-ONC1).

In this follow-up work to our study concerning canine tumors [8], we are aiming at establishing a way to quickly determine the effectiveness of GLV-1h68-treatment against two different types of human tumors, namely hepatocellular carcinoma (HCC) and prostate carcinoma. These tumor types belong to the most common malignancies worldwide [9–11]. To this end we will use immune-related antigen profiling data from tumor samples of xenografted nude mice in order to determine a distinct signaling pattern for each of the tumor types. We will also compare the protein antigen profiling of GLV-1h68-infected female or male PC-3 xenografted mice.

In this paper we will strengthen the thesis that our Boolean model could be a useful for testing and optimizing of OV-mediated cancer therapy already before its application in patients.

## Results

To estimate the effectiveness of VACV-treatment *in silico* of the different tumor types (PLC, HuH7 and PC-3) we obtained accurate immune-related protein antigen profiles (Supplementary table 1). These were taken 10 or 42 days after vaccinia virus GLV-1h68 or PBS treatment. Mice from each group were sacrificed and mouse immune-related protein antigen profiles were generated. The antigen profiling data were used to calculate correlates of antigen quantities before and after treatment with VACV. These correlates where then directly applied as coefficients of input signal strengths for the input nodes of the model.

*In silico* effects are similar to *in vivo* effects – although the calculated signal strength for tumor apoptosis *in silico* is not equal to the observed remission of tumor size *in vivo*: the calculation only gives information if the tumor goes into remission, but there is no information as to how fast this happens *in vivo.*

**Figure 1.**
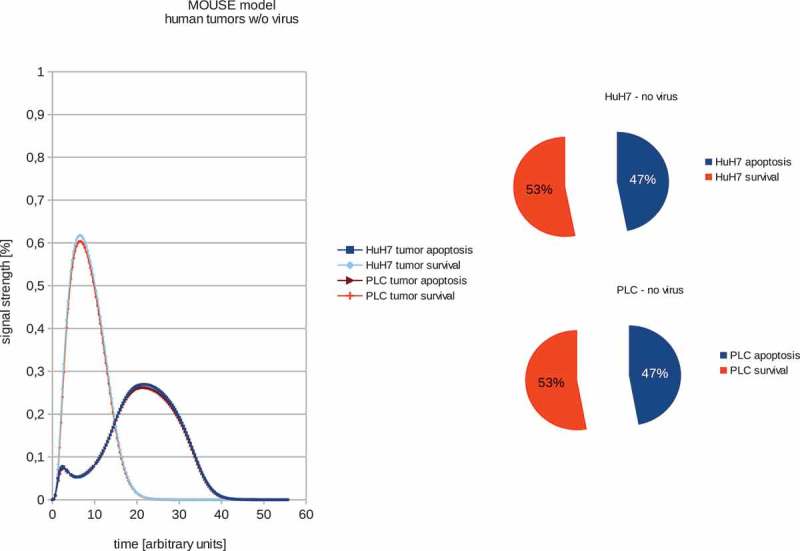
Models and ratios of tumor survival and apoptosis of the human PLC and HuH7 tumors in female nude mice. In order to estimate the differences between tumor survival and apoptosis, the polynomial area of the diagrams was calculated. Without viral therapy the tumors are in indefinite proliferation.

**Figure 3.**
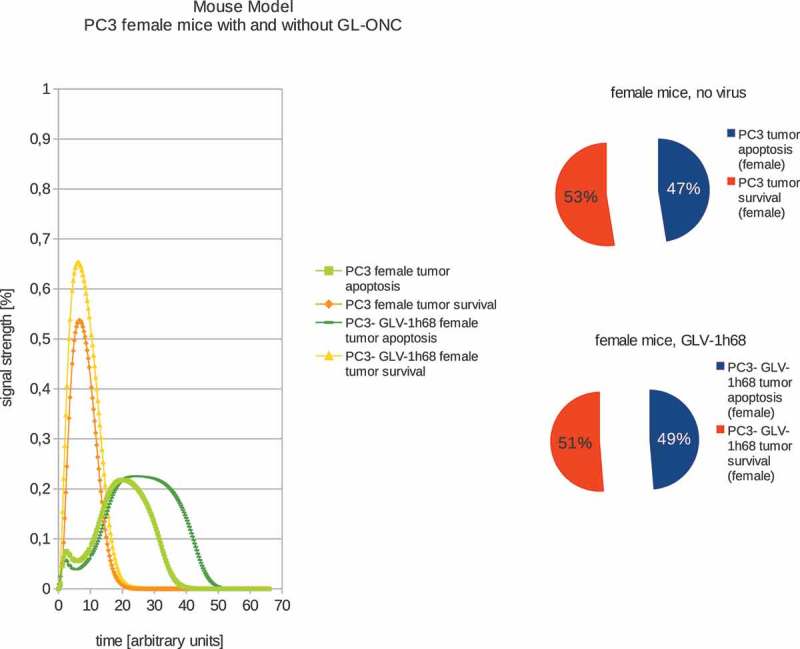
Models and ratios of tumor survival and apoptosis of the human PC-3 tumors in female nude mice without and with GLV-1h68-treatment. Without oncolytic virus therapy the tumor apoptosis rate (53%) is was set to be higher than the apoptotic rate (47%) and therefore simulating a proliferating tumor. After calculating the effects of viral therapy, these values ended up at 51% tumor survival and 49% tumor apoptosis and therefore ensuring tumor survival, albeit to a lesser extent.

**Figure 4.**
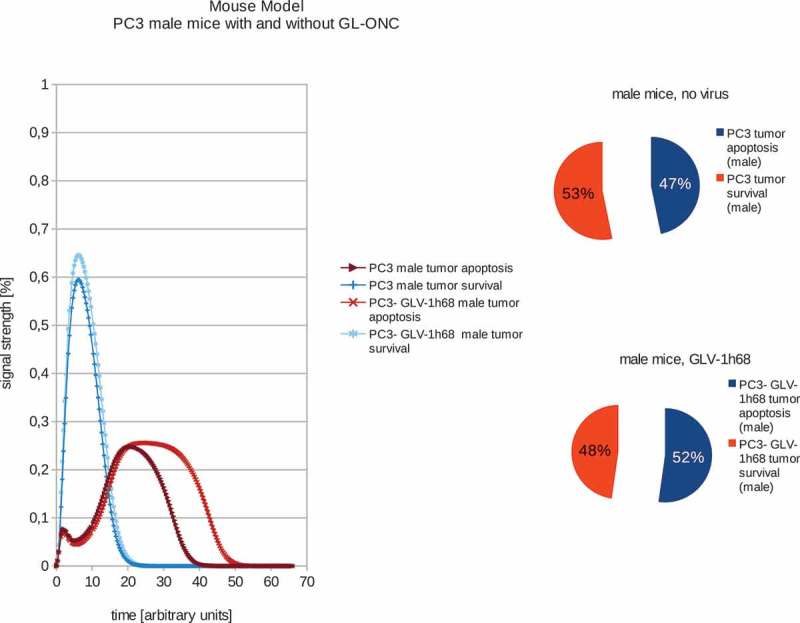
Models and ratios of tumor survival and apoptosis of the human PC-3 tumors in male nude mice without and with GLV-1h68-treatment. In stark contrast to the female mice, tumors in male mice react very differently to oncolytic virus therapy: after application of GLV-1h68 the tumor survival rate was calculated to be 48% with an apoptosis rate of 52% and therefore indicating tumor apoptosis.

In order to train the models to the *in vivo* findings we set the proliferation rate – in the case of non-treated tumors – to always be higher than the tumor remission (see Figures 1–4). These fitted models were set to have a proliferation signal strength of 53% and 47% apoptosis signaling strength accordingly. These values were chosen to be well above/below the maximum standard error of the calculations of 1.5%. The boolean signaling model, as previously described by Cecil et al [8]., was then used to calculate the differences in the response of the different tumor types to VACV. In order to estimate the differences between tumor survival and apoptosis, the polynomial area of the diagrams was calculated. Resulting boolean signaling strength of the networks depending on correlations of tumor growth and remission in human tumors with and without viral treatment show that PLC, HuH7 and PC-3 tumors are all affected by VACV-treatment.

After mapping the distinct signals after viral treatment and emulation of the innate immune systems response to tumorous cells, all tested tumor types are in remission *in silico* (Figures 1–4). Boolean modeling correctly emulates *in vivo* findings to GLV-1h68 treatment [12–14].

In addition, we also calculated the effects of GLV-1h68-treatments in female or male PC-3 tumor xenografted mice (Figures 3,4). Our data demonstrated that in male mice, GLV-1h68 injection led to more efficient apoptosis than in female mice bearing PC-3 tumors. However, there are no significant differences between female and male PC-3 xenografts after virus injection. These boolean modeling data correlate very well with the data of tumor regression found in both male and female PC-3 xenografts after virus injection [13].

## Discussion

In this work we establish that boolean modeling is not only suitable for calculating the effects of VACV-treatment on tumor growth in canine xenograft models [8], but also for human tumors. Human cancer patients could benefit from these models as we are now able to predict the tumor dependent outcome of the VACV-therapy according to antigen profiling data, which can directly be fitted to the models and correctly predict the outcome of VACV-therapy.

In all analyzed virus-infected tumors in this study, the profiling of proteins related to immune response revealed a significant proinflammatory immune response and marked activation of innate immunity in tumor microenvironment. This change seems to correspond well with the tumor regression. Similar data also have been obtained in xenograft models of human pancreatic [6] and human lung adenocarcinomas [15] suggesting that it is a general mechanism of the vaccinia virus GLV-1h68 to induce strong innate host immune responses in tumors of different origins. The presence of such activated inflammatory cells in the tumor tissue could enhance the anti-tumoral effect by increasing the phagocytic or cytotoxic activities of these cells [16,17]. In addition, the xenograft models could provide important information about the Vaccinia virus infectivity of the tested tumor types. Interestingly, GLV-1h68 was also detected in livers of male and female PC-3 xenografted mice at day 42 after virus injection [13]. In this case, the later presence of GLV-1h68 in this organ might also be a mark for metastases formation. Moreover, we have demonstrated that GLV-1h68 is a highly tumor- and metastases-selective in this PC-3 model [13,14].

In future work we want to expand these predictive qualities by building tumor response libraries for later use in patients. These libraries will contain antigen profiling data before and after VACV-treatment, treatment regimen and treatment outcome. From there, we will then match the antigen profiling data before and after treatment and find the common denominators for positive or negative treatment outcome with different virus strains. In the end we will be able to quickly analyze a patient antigen profile before treatment and devise the most promising treatment regimen for this patient.

Certain limitations to this approach apply: we focused solely on signaling and had to deal with limited data, in particular, detailed kinetics were not available. Due to limited data available, the semi-quantitative approach was the only feasible mode to calculate interpolations between full ON or OFF states of signaling nodes by e-functions.

By collecting more information about the specific signal strengths of key players in the apoptosis signaling networks we also want to improve our modeling of the regulatory effects of these key players. Numerous studies are available describing apoptosis pathways and modifications. It has to be, however, noted that the alteration of apoptotic signaling cascades via different virus strains is a rather complex undertaking. It could, however, be considered for oncolytic viruses in an iterative combination of *in* silico modeling and experimental data collection to narrow down the possible pathways which should be considered first as possible oncolytic virus targets.

## Materials and methods

### Ethics statement

All animal experiments were carried out in accordance with protocols approved by the Institutional Animal Care and Use Committee (IACUC) of Explora Biolabs (San Diego, CA, USA; protocol number: EB11-025) and/or the government of Unterfranken, Germany, according to the German Animal Welfare Act (TierSchG) (permit number: 55.2–2531.01–17/08).

### Cancer cell lines

The two human hepatocellular carcinoma cell lines HuH7 (ATCC CCL-185) and PLC/PRF/5 (PLC; ATCC CRL 8024) were obtained from the American Type Culture Collection (ATCC). Cells were cultured in Dulbecco’s modified Eagle’s medium (DMEM) supplemented with antibiotics (100 units/ml penicillin G, 100 units/ml streptomycin) and 10% fetal bovine serum (FBS; Invitrogen GmbH, Karlsruhe, Germany) at 37°C under 5% CO_2_.

The human prostate carcinoma cell line PC-3 (DSMZ ACC465) was cultured in RPMI 1640 (PAA Laboratories, Cölbe, Germany) supplemented with 10% FCS (PAA Laboratories, Cölbe, Germany) and 1% penicillin-streptomycin solution (PAA Laboratories, Cölbe, Germany) at 37°C under 5% CO_2_.

### Vaccinia virus strain GLV-1h68

GLV-1h68 was developed from a Lister virus strain by inserting three expression cassettes encoding *Renilla* luciferase–*Aequorea* green fluorescent protein (Ruc-GFP) fusion protein, LacZ, and β-glucuronidase into the *F14.5L, J2R* (thymidine kinase) and *A56R* (hemagglutinin) loci of the viral genome, respectively [5].

### Vaccinia virus-mediated therapy of human xenografts

Tumors were generated by implanting human hepatoma cells HuH7 or PLC (5 x 10^6^ cells) or human prostate carcinoma cells PC-3 (2 x 10^6^ cells) subcutaneously on the right flank above the hind leg of 6- to 8-week-old female or male nude mice (NCI/Hsd/Athymic Nude-Foxn1nu, Harlan Winkelmann GmbH, Borchen, Germany). On day 10 post implantation, a single dose of the GLV-1h68 virus (5 x 10^6^ plaque forming units [pfu] in 100 ml PBS) was injected into the tail vein (i.v.) of HuH7, PLC or PC-3 tumor-bearing mice. The control animals were injected i.v. with PBS only.

The vaccinia virus GLV-1h68 strain-mediated effects on the tumor growth of PLC, HuH7 and PC-3 xenografts were already described [12–14].

### Antigen profiling data

At 10 or 42 days after virus or PBS treatment, three mice from each group were sacrificed. Tumors were excised, weighted, and homogenized using FastPrep FP120 Cell Disruptor (BIO 101, Qbiogene, Germany) at a speed of 6,000 x g for 20 s (three times). Samples were then centrifuged at 10,000 x g at 4°C for 5 min and the supernatants were analyzed for mouse immune-related protein antigen profiles by Rules Based Medicine (Austin, USA) via mouse Multi-Analyte Profiles using antibody linked beads. Results were normalized based on total protein concentration.

### Dynamic modeling

The most relevant antigens from the general apoptosis and tumor survival signaling networks were identified, as well as key molecular components for all considered cascades were incorporated into a machine readable CellDesigner [18] network model, which takes their respective logical connectivity into account. In order to present the model in a more appealing way, we refined the layout in the yED- Graph Editor [19]. It includes the apoptosis, MAPK, p53, WNT, Hedgehog, TK cell mediated cell death, IFN and Interleukin signaling, as well as mitochondrial Ca^2+^-signaling (see supplementaryable 1 for details). Custom scripting for boolean semi-quantitative modeling in SQUAD [20] was then set up using the topology information and logical connectivity from the CellDesigner model. Semi-quantitative modeling is reached by the SQUAD-algorithm applying e-functions to interpolate between ‘ON’ and ‘OFF’ boolean states. These simulations model the effects of different inputs into the model and the subsequent response of the system [8].

Antigen profiling data was obtained for PLC, HuH7 and PC-3 tumors with and without viral treatment. Input data for SQUAD-modeling consists of Interleukin, TNF-α and IFN-γ antigens.

To simulate signaling networks from antigen quantity, the differences in measured antigen quantities were compared before and after viral treatment. The resulting coefficient was then used to define the starting signal strength in the models. The models were also calibrated to simulate tumor proliferation in non-treated tumor cells. For tumor cells which underwent viral treatment, the same calibration as in the non-treated cells was used, but the signaling of natural killer cells was, additionally to the different signaling inputs derived from antigen quantity, activated to emulate the effects of the innate immune system of nude mice on the tumor cells.

Differences in signal strength leading to tumor growth or remission were directly compared as well as by their integrals.

## Supplementary material

The supplemental data for this article can be accessed here.
