# Supplementary material for: Vaccinia virus injected human tumors: oncolytic virus efficiency predicted by antigen profiling analysis fitted boolean models
Source: Bioengineered. 2019 May 29;10(1):190–6. doi: 10.1080/21655979.2019.1622220 (PMC6550548; doi:10.1080/21655979.2019.1622220)
Supplement: Supplemental Material [file kbie-10-01-1622220-s001.zip › supplementary information/Supplementary legend.docx]

**Supplementary tables:**

Supplementary table1:

This table contains all antigen immunoassays as well as the calculated ratios for all the different tested tumor models before and after oncolytic therapy. These ratios form the starting point for the *in silico* models described in this work.

Supplementary table 2:

This table contains the calculations for the survival and apoptosis rates of all tested tumor strains.
